# Left atrial vortex flow and its relationship with left atrial functions in patients with congenital heart disease

**DOI:** 10.1186/s43044-024-00486-2

**Published:** 2024-05-02

**Authors:** Keita Ito, Hideharu Oka, Yuki Shibagaki, Yuki Sasaki, Rina Imanishi, Sorachi Shimada, Yuki Akiho, Kazunori Fukao, Sadahiro Nakagawa, Kunihiro Iwata, Kouichi Nakau, Satoru Takahashi

**Affiliations:** 1 Department of Pediatrics, Asahikawa Medical University, 2-1-1-1, Midorigaoka-Higashi, Asahikawa, Hokkaido 078-8510 Japan; 2 Section of Radiological Technology, Department of Medical Technology, Asahikawa Medical University Hospital, Asahikawa, Hokkaido Japan

**Keywords:** 4D flow MRI, Left atrial vortex flow, Left atrial function, Reservoir strain

## Abstract

**Background:**

Four-dimensional flow magnetic resonance imaging (MRI) enables blood flow visualization. The absence of left atrial vortex flow (LAVF) has been implicated in the development of thrombus formation and arrhythmias. However, the clinical relevance of this phenomenon in patients with congenital heart disease (CHD) remains unclear. This study aimed to unravel the relationship of LAVF with left atrial functions in patients with CHD.

**Results:**

Twenty-five participants who underwent cardiac MRI examinations were included (8 postoperative patients with CHD aged 17–41 years and 17 volunteers aged 21–31 years). All participants were in sinus rhythm. Four-dimensional flow MRI (velocity encoding 100 cm/s) assessed the presence of LAVF, and its relationship with left atrial function determined by transthoracic echocardiography was explored. LAVF was detected in 16 patients. Upon classification of the participants based on the presence or absence of LAVF, 94% of participants in the LAVF group were volunteers, while 78% of those in the without LAVF group were postoperative patients. Participants without LAVF had a significantly lower left atrial ejection fraction (61% vs. 70%, *p* = 0.019), reservoir (32% vs. 47%, *p* = 0.006), and conduit (22% vs. 36%, *p* = 0.002) function than those with LAVF.

**Conclusions:**

LAVF occurred during the late phase of ventricular systole, and left atrial reservoir function may have contributed to its occurrence. Many postoperative patients with CHD experienced a loss of LAVF. LAVF may indicate early left atrial dysfunction resulting from left atrial remodeling.

## Background

Left atrial performance is associated with arrhythmias and heart failure. Reduced left atrial performance has been linked to an elevated incidence of atrial flutter and the progression of pulmonary congestion due to left heart failure [1]. Echocardiography is a prevalent technique used to evaluate left atrial function, and left atrial reservoir strain is an informative parameter for assessing left atrial performance and remodeling [2]. Recently, blood flow dynamics at various sites have been elucidated by four-dimensional (4D) flow magnetic resonance imaging (MRI), which has revealed the presence of vortex flow in the left atrium of healthy adults [3]. Blood flows from the pulmonary vein into the left atrium during early ventricular systole, resulting in vortex flow in the left atrium during late ventricular systole, and thereafter flows out to the left ventricle during early ventricular diastole [3]. The purported role of left atrial vortex flow (LAVF) is to maintain stable blood flow, thereby preventing energy loss and optimizing the coupling of atrium and ventricle [4]. It has been reported that increased left atrial volume, decreased flow velocity during left atrial contraction, and blood flow imbalance from the left and right pulmonary veins cause loss of LAVF, which can lead to thrombus formation and arrhythmias [3]. As many patients with congenital heart disease (CHD) have survived into adulthood with improved treatment outcomes, various postoperative remote-phase complications have emerged [5]. Numerous studies have been conducted to explore the correlation between left atrial functionality, left ventricular diastolic capacity, and life expectancy in patients with CHD [6–8]. Nevertheless, no study has investigated LAVF in postoperative patients with CHD, and its clinical significance remains unclear. Therefore, this study aimed to analyze LAVF using 4D flow MRI and to examine its relationship with left atrial function.

## Methods

**Fig. 1:**
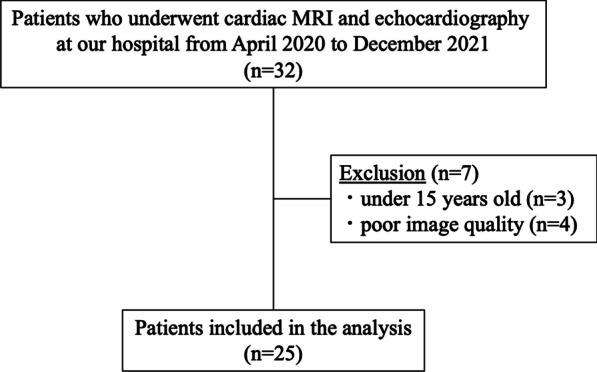
Patient flow

A total of 32 participants who underwent cardiac MRI examinations at Asahikawa Medical University Hospital between April 2020 and December 2021 were enrolled in the study (Fig. 1). Following the acquisition of written informed consent from all participants or their parents, 4D flow MRI was conducted within 3 days of the cardiac ultrasound examination.

All participants underwent cardiovascular MRI with a 3.0 Tesla whole-body imager (MAGNETOM Vida 3 T; Siemens Healthcare, Erlangen, Germany). Scan parameters were as follows: acceleration method, GRAPPA 3; field of view, 340 × 340 mm; and flip angle, 8°. Four-dimensional flow acquisitions were free breathing, using a prospectively electrocardiogram-triggered, free-breathing cardiorespiratory-synchronized, three-dimensional, three-directional, time-resolved phase-contrast MRI sequence with a 41ms measurement temporal resolution. The acquired voxel size was 3.6 × 3.6 × 3.6 mm^3^ (reconstructed voxel size: 3.6 × 3.6 × 1.8 mm^3^), with velocity encoding of 100 cm/s, segment 1, and > 25 frames/cycle. A workstation (Cvi42, Circle, Cardiovascular Imaging, Calgary, Canada) was used for the analysis. The presence of LAVF was qualitatively assessed by a pediatric cardiologist with over a decade of experience.

Following acquisition of the apical four-chamber view on transthoracic echocardiography, left atrial strain analysis was conducted on the same section. The QRS wave of the electrocardiogram was designated as the initial point of strain, and strain values related to reservoir, conduit, and contractile function were calculated. Additionally, left atrial ejection fraction (LAEF, %) and left atrial end-diastolic volume (LAEDV, mL) were computed. Image analysis was conducted with ViewPal (GE Medical System, Horten, Norway).

All parameters are expressed as median and range. Statistical analyses were conducted using SPSS 25.0 (IBM SPSS Inc, Chicago, IL, USA). We confirmed the normal distribution of the data with the Shapiro–Wilk test. After testing for homogeneity of variances with Levene's test, we compared the two groups with independent T-tests. A *p*-value of < 0.05 was considered to be statistically significant. This study was conducted in compliance with the standards of the Declaration of Helsinki and the current ethical guidelines and was approved by our institutional ethics board (Approval Number 19250). Written informed consent was obtained from all the participants.

## Results

**Fig. 2:**
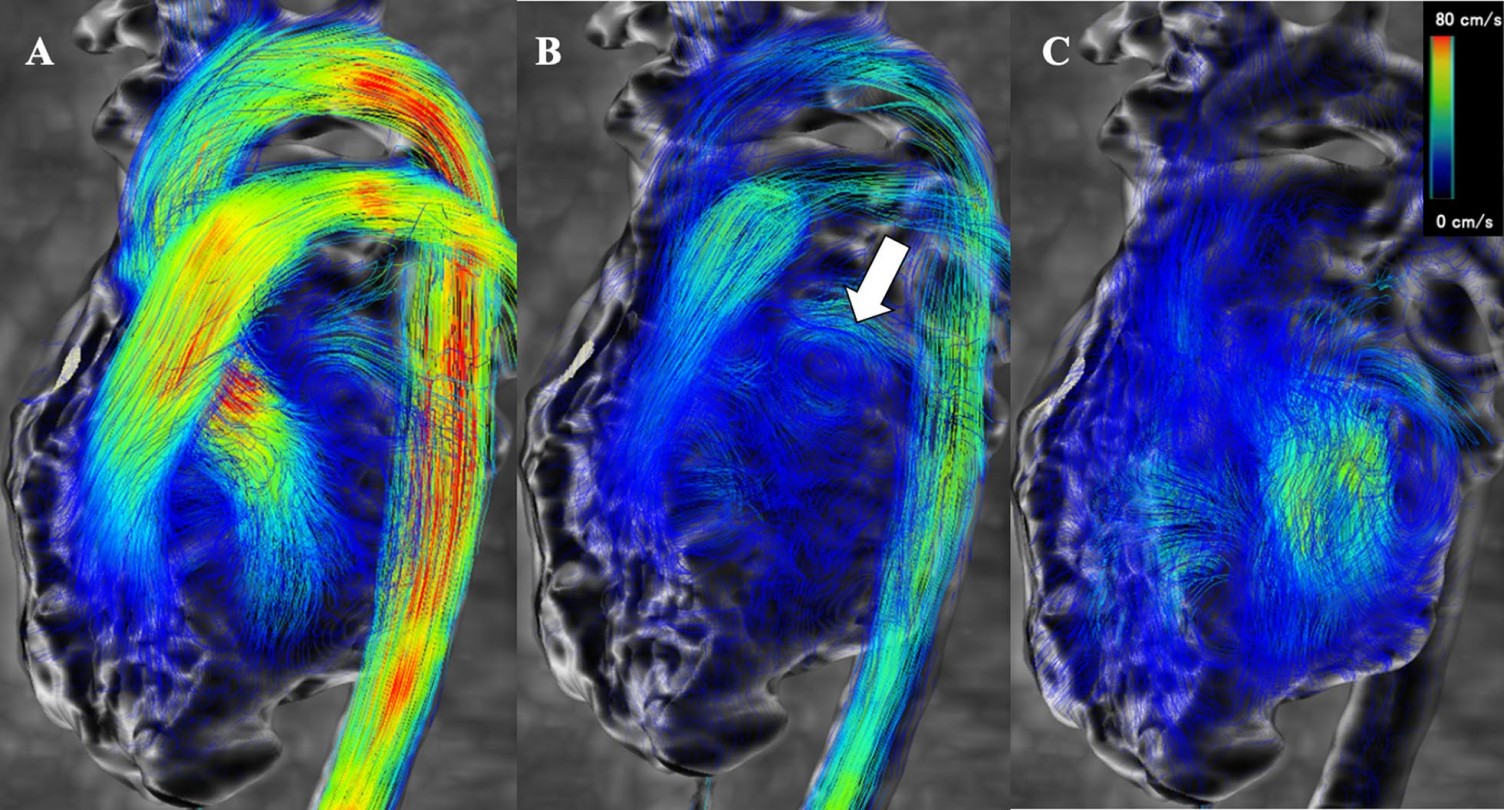
MRI images depicting left atrial vortex flow throughout a single cardiac cycle in a healthy individual are presented. **A** Pulmonary venous blood enters the left atrium in the early phase of ventricular systole, **B** Left atrial vortex flow (white arrow) transpires in the late phase of ventricular systole, **C** Blood flows into the left ventricle during the early phase of ventricular diastole

To avoid the influence of age on LA function, 3 participants were excluded who were under 15 years old. Four participants were excluded due to the poor image quality of 4D flow MRI. Of the 25 remaining participants, 8 were postoperative patients with CHD (3 male individuals, aged 17–41 [median: 22] years) and 17 were healthy volunteers (11 male individuals, aged 21–31 [median: 23] years). Echocardiography was performed to exclude any cardiovascular pathology in the healthy subjects. The postoperative CHD cohort included three patients with tetralogy of Fallot, two with complete transposition of the great arteries, one with total anomalous pulmonary venous return, one with double outlet right ventricle, and one with pulmonary artery stenosis. The demographic data of the participants and the prevalence of LAVF are summarized in Table 1. All individuals were in sinus rhythm during 4D flow MRI and echocardiography, and none exhibited arrhythmias. The median hemoglobin was 14.7 g/dL and the median NT-proBNP was 24.2 pg/mL, and none of the patients showed obvious polycythemia or left ventricular dysfunction. Figure 2 shows LAVF imaged by 4D flow MRI. LAVF was observed in 16 of 25 (64%) participants, with only one (13%) in the CHD group and 15 (88%) in the volunteer group demonstrating its presence. Subsequently, the participants were classified into two distinct groups based on the presence or absence of LAVF, as outlined in Table 2. There were no significant differences in age, sex, or height between the two groups. Although body weight and body surface area (BSA) were substantially higher in the group that lacked LAVF, there was no significant difference in the LAEDV/BSA between the two groups (no vortex vs. vortex: LAEDV/BSA 19.4 vs. 26.7 mL/m^2^, *p* = 0.374). The group without LAVF had a notably lower LAEF (61% vs. 70%, *p* = 0.019), reservoir (32% vs. 47%, *p* = 0.006), and conduit (22% vs 36%, *p* = 0.002) function than that with LAVF. However, there was no significant difference between the groups in contractile function (8% vs. 11%, *p* = 0.737).

**Table 1:** Demographic data

	Volunteers (*n* = 17)	CHD (*n* = 8)	TOF	TOF	TOF	TGA	TGA	TAPVR	DORV	PS
Age (years)	23 (21–31)	22 (17–41)	31	20	17	36	19	17	24	41
Male: Female	11: 6	3: 5	M	M	F	F	M	F	F	F
Height (m)	1.67 (1.48–1.8)	1.63 (1.52–1.77)	1.74	1.64	1.65	1.6	1.77	1.62	1.58	1.52
Body weight (kg)	57 (45–74)	71 (42.3–112.2)	70.7	53.5	54.9	75.7	89.2	112.2	70	42.3
BSA (m^2^)	1.57 (1.42–1.89)	1.79 (1.36–2.25)	1.85	1.56	1.59	1.83	2.09	2.25	1.75	1.36
Hemoglobin (g/dL)	–	14.7 (13–16.5)	13	16.5	14.7	13.6	15.1	16.2	14.7	13.7
NT-proBNP (pg/mL)	–	24.2 (6.7–139)	115	33.9	21.4	23.6	6.7	17.8	24.7	139
Surgical procedures	–	–	LMBTS at the age of 2 months, RMBTS at the age of one year, ICR at the age of 5 years	ICR at the age of 18 months	ICR at the age of 3 months	ASO at the age of 6 months	ASO at the age of 13 days	ICR at the age of one month	ICR at the age of 8 months	ICR at the age of one month
Left atrial vortex flow (+)	15	1	(−)	(−)	(−)	(−)	(−)	(−)	(−)	(+)

All parameters are expressed as median and range

*BSA* body surface area; *NT-proBNP* N-terminal prohormone of brain natriuretic peptide; *CHD* congenital heart disease; *TOF* tetralogy of Fallot; *TGA* transposition of the great arteries; *TAPVR* total anomalous of pulmonary venous return; *DORV* double outlet right ventricle; *PS* pulmonary valve stenosis; *LMBTS* left modified Blalock–Taussig shunt; *RMBTS* right modified Blalock–Taussig shunt; *ICR* intracardiac repair; *ASO* arterial switch operation

**Table 2:** Comparison between left atrial vortex flow and its absence

	Left atrial vortex flow		*p* value
	( +)	( −)	
	(*n* = 16)	(*n* = 9)	
Age (years)	23.5 (22–41)	22 (17–36)	0.525
Male: Female	10: 6	4: 5	0.404
Height (m)	1.68 (1.52–1.8)	1.64 (1.48–1.77)	0.302
Body weight (kg)	54 (42–74)	70 (50–112)	0.013
BSA (m^2^)	1.57 (1.34–1.89)	1.75 (1.43–2.25)	0.042
Participants			
CHD	1	7	
Volunteers	15	2	
LAEF (%)	70 (52–85)	61 (42–75)	0.019
LAEDV/BSA (mL/m^2^)	26.7 (15–41.6)	19.4 (12.5–29.2)	0.374
Reservoir (%)	47 (26–62)	32 (15–42)	0.006
Conduit (%)	36 (20–51)	22 (12–33)	0.002
Contractile (%)	11 (4–14)	8 (1–22)	0.737

All parameters are expressed as median and range

*BSA* body surface area; *CHD* congenital heart disease; *LAEF* left atrial ejection fraction; *LAEDV* left atrial end-diastolic volume

## Discussion

This is the first report on the assessment of LAVF utilizing 4D flow MRI in individuals with CHD. We investigated the correlation between LAVF and left atrial function among postoperative patients with CHD and discovered two findings. First, compared with healthy participants, a greater number of postoperative patients with CHD exhibited a lack of LAVF. Second, patients with a loss of LAVF demonstrated lower left atrial ejection fraction, reservoir, and conduit function.

A significant number of postoperative patients with CHD had no LAVF. Suwa et al. conducted 4D flow MRI and transthoracic echocardiography in 32 participants and disclosed that patients devoid of LAVF had significantly more heart disease [3]. Although the patients in that study had heart diseases that were different from those in the present study, such as myocardial infarction, takotsubo cardiomyopathy, aortic regurgitation, and hypertensive heart disease, the results were similar in that LAVF vanished in individuals with heart disease. Although the clinical significance of LAVF remains unclear, Park et al. reported that it may serve to avert intra-atrial blood stasis [9]. Postoperative patients with CHD are more likely to develop arrhythmias, which are caused by congenital abnormalities of the conduction system, acquired hemodynamic abnormalities due to pressure and volume loading, conduction system abnormalities, and myocardial damage complicated by hypoxia, surgery, and aging [1]. A loss of LAVF may increase the risk of intra-atrial thrombus formation due to stasis of blood flow and arrhythmias, such as atrial fibrillation.

**Fig. 3:**
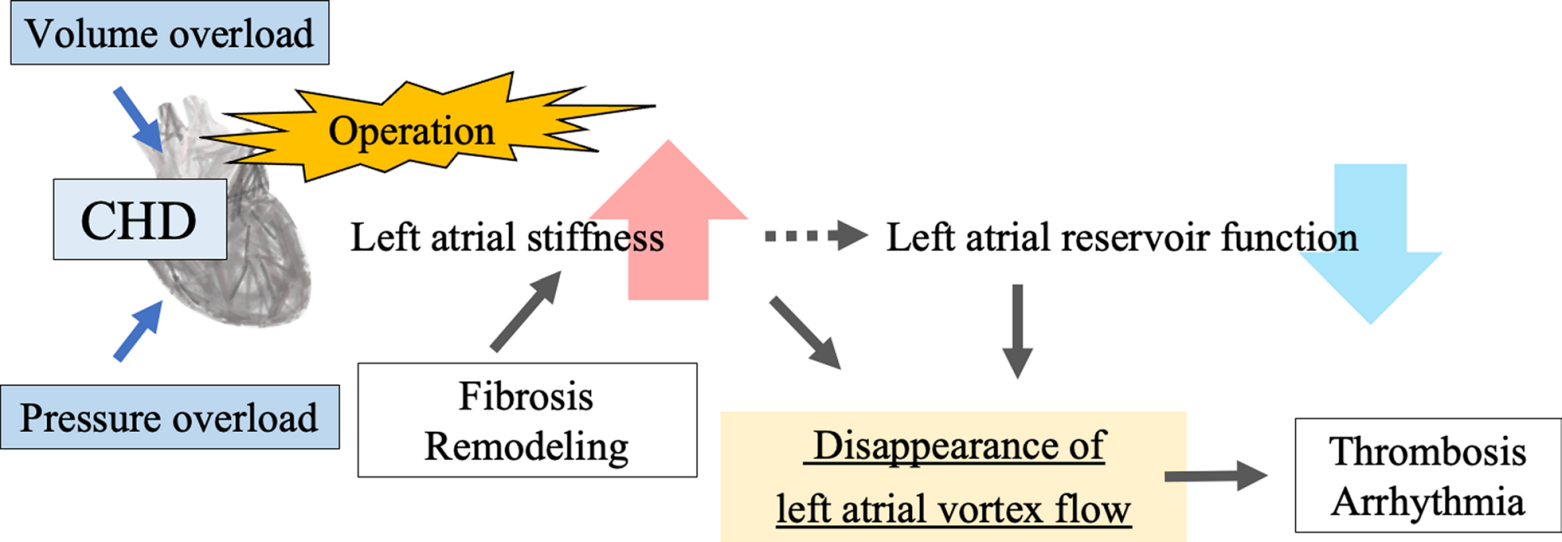
The mechanics underlying left atrial vortex flow in patients with postoperative congenital heart disease (CHD) are demonstrated. In such individuals, surgical intervention leads to the development of fibrosis and remodeling, which subsequently results in augmented left atrial stiffness, pressure, and volume loading. It is hypothesized that this heightened stiffness diminishes left atrial reservoir function, ultimately leading to the disappearance of left atrial vortex flow

The present study observed that patients with a loss of LAVF exhibited a decline in left atrial ejection fraction, reservoir, and conduit function. Considering that LAVF typically manifests during the late phase of ventricular systole, also known as late atrial diastole, it was hypothesized that the decline in reservoir function was closely linked to the loss of vortex flow. Patients with CHD may display augmented left atrial stiffness as a result of fibrosis and remodeling due to pressure and volume loading, as well as surgical intervention [10–12]. Consequently, the left atrial reservoir function was reduced, and the LAVF vanished (Fig. 3) [13]. Left atrial reservoir function is an important determinant of cardiac output and is among the prognostic factors for diastolic dysfunction [14, 15]. In this current investigation, LAVF was absent in patients with a left atrial reservoir function of ≤ 25%. Nonetheless, some patients with a left atrial reservoir function of > 25% also showed a loss of LAVF, implying that the absence of LAVF may be an early sign of left atrial dysfunction. If the absence of LAVF results from left atrial remodeling, it may also serve as a valuable predictor of future arrhythmias. Therefore, patients with absent LAVF may be closely monitored for potential thrombus formation and arrhythmias.

The limitations of this study are as follows. First, this was a single-center study, which restricted the number of evaluators who measured LAVF and function. Second, the sample size was significantly small. To generalize these findings, a large number of cases must be included in a large-scale prospective study. Third, there was no direct comparison between left atrial fibrosis or stiffness and vortex flow. Cardiac MRI is often inconclusive in diagnosing left atrial fibrosis while being challenging to quantify this condition. However, left atrial stiffness can be approximated using the results of cardiac catheterization, and we are considering conducting future studies.

## Conclusions

In conclusion, many postoperative patients with CHD experienced a loss of LAVF. LAVF may indicate early left atrial dysfunction resulting from left atrial remodeling, thereby facilitating early intervention and favorable outcomes.

## Data Availability

Raw data were generated at Asahikawa Medical University. Derived data supporting the findings of this study are available from the corresponding author H.O. on request.

## Abbreviations

BSA: Body surface area
CHD: Congenital heart disease
4D: Four-dimensional
LAEF: Left atrial ejection fraction
LAEDV: Left atrial end-diastolic volume
LAVF: Left atrial vortex flow
MRI: Magnetic resonance imaging
